# What Are Fair Study Benefits in International Health Research? Consulting Community Members in Kenya

**DOI:** 10.1371/journal.pone.0113112

**Published:** 2014-12-03

**Authors:** Maureen Njue, Francis Kombe, Salim Mwalukore, Sassy Molyneux, Vicki Marsh

**Affiliations:** 1 Kenya Medical Research Institute (KEMRI) - Wellcome Trust Research Programme, PO Box 230, Kilifi, 80108, Kenya; 2 Centre for Tropical Medicine and Global Health, Nuffield Department of Medicine Research Building, Oxford University, Old Road Campus, Headington, Oxford, OX3 7FZ, United Kingdom; 3 Ethox Centre, Nuffield Department of Population Health, Oxford University, Old Road Campus, Headington, Oxford, OX3 7LF, United Kingdom; University of California, San Francisco, United States of America

## Abstract

**Background:**

Planning study benefits and payments for participants in international health research in low- income settings can be a difficult and controversial process, with particular challenges in balancing risks of undue inducement and exploitation and understanding how researchers should take account of background inequities. At an international health research programme in Kenya, this study aimed to map local residents' informed and reasoned views on the effects of different levels of study benefits and payments to inform local policy and wider debates in international research.

**Methods and Findings:**

Using a relatively novel two-stage process community consultation approach, five participatory workshops involving 90 local residents from diverse constituencies were followed by 15 small group discussions, with components of information-sharing, deliberation and reflection to situate normative reasoning within debates. Framework Analysis drew inductively and deductively on voice- recorded discussions and field notes supported by Nvivo 10 software, and the international research ethics literature. Community members' views on study benefits and payments were diverse, with complex contextual influences and interplay between risks of giving ‘too many’ and ‘too few’ benefits, including the role of cash. While recognising important risks for free choice, research relationships and community values in giving ‘too many’, the greatest concerns were risks of unfairness in giving ‘too few’ benefits, given difficulties in assessing indirect costs of participation and the serious consequences for families of underestimation, related to perceptions of researchers' responsibilities.

**Conclusions:**

Providing benefits and payments to participants in international research in low-income settings is an essential means by which researchers meet individual-level and structural forms of ethical responsibilities, but understanding how this can be achieved requires a careful account of social realities and local judgment. Concerns about undue inducement in low-income communities may often be misplaced; we argue that greater attention should be placed on avoiding unfairness, particularly for the most-poor.

## Background

In biomedical research involving people, payments to research participants - in cash or in kind - have long been routine practice. They have also long generated controversy on ethical issues associated with levels and types of payments and their relationship to the contexts in which research is conducted. The giving of cash or ‘in kind’ payments to study participants has been justified in a number of ways. Least or non- controversially, they are given to compensate for time, inconvenience and other burdens experienced by participants, and to reimburse any direct or indirect costs incurred so that research participants are not made ‘worse off’ by their participation [1], [2]. This principle underpins much current guidance on cash payments for reimbursement of fares in travelling to research clinics, or time lost from paid employment.

Many aspects of payments have remained ethically controversial [3], including their use in supporting recruitment – as a *due* incentive - and in appreciating participants' contributions to research [4]. The main concern has been the potential for cash or in kind payments to introduce an *undue* form of inducement, leading to ‘clouding’ of individual judgment, the taking of unnecessary, unreasonable or excessive risks of physical and non-physical harms [5] and a related impairment of voluntariness in decision-making. Inducements are seen as problematic if they encourage falsification of data where potential participants are keen to ‘qualify’ for recruitment, with potential implications for participants' safety and the validity of research [4], [6], [7]. Particular concerns have been expressed for research in low-income settings, where relatively moderate levels of benefits and payments might generate ‘undue’ inducement, and the most-poor might bear a disproportionate burden of research [3], [4]. Additional concerns about benefits and payments are their potential to commercialise the relationship between investigators and participants, undermining altruism; and the impact of the costs of payments on researchers' and funders' capacity to undertake research of high social value [3].

The main dilemma introduced by limiting payments as a response to these challenges is the risk of ‘exploitation’ of participants. As summarised by Ballantyne (2008): *‘offer participants too little and they are exploited, offer them too much and their participation may be unduly induced’* (p 179) [8]. Inclusive in the broad idea of ‘exploitation’ in the literature are concerns that offering ‘too few’ benefits and payments to the most-poor might make research inaccessible or unattractive to these groups, such that they do not benefit from the immediate or long term gains of studies; that failure to include the most-poor would bias the results of studies; and that this would limit the options of already vulnerable populations - an unwarranted form of paternalism - whereas researchers should recognise a responsibility to protect this group [3], [4].

The concerns outlined so far are closely related to debates on the ethical responsibilities of international health researchers working in low-income settings for study benefits in general. In recent years there has been increasing emphasis on the importance of researchers recognising *macro-level* or structural (such as poverty and lack of access to health care) as well as *micro-level* or individual ethical issues for participants (such as ensuring voluntary informed consent and balancing risks and benefits of participation in particular studies) when considering study benefits [9]–[11]. The importance of both macro- and micro-level ethical issues in these settings underpins broad agreement on a Fair Benefits Approach [6], [12], [13] in which a range of benefits and beneficiaries are seen as important, rather than only benefits due to individual study participants in relation to the costs of a given study. This broader concept of benefits includes study- specific and more widely research-related benefits to participants and the general community, including medical benefits, other goods or payments acting as benefits, employment opportunities, capacity-building for health care provision and science, and support to long term health care delivery. Within this literature there remains disagreement on a normative account of ‘fairness’ in Fair Benefits, but agreement on a role for local voices in planning.

Current national and international guidelines for the ethical conduct of research offer little operational guidance on how to arrive at appropriate levels and types of study benefits and payments. Instead, recognising the importance of informing these issues in a specific context, many guidelines recommend planning through local forms of consultation [1], particularly for international research in low-income settings. In an earlier publication, we described some common practices in giving study benefits and payments at an international research programme in Kenya and the views of a range of research staff on the appropriateness of these [14]. The current paper reports on an empirical study undertaken at the same research programme to explore the views of local residents in the area around the research programme on how study benefits and payments should be planned, including cash payments, and medical, non-medical and community-wide benefits. The paper aims to support the development of local guidelines and contribute to the wider debate in the literature.

## Methods

### 2.1 Study site

This study was conducted in the geographic area surrounding the Kenya Medical Research Institute (KEMRI) Wellcome Trust Research Programme in Kilifi, a long-established international health research programme in Kenya, described in detail elsewhere [15]. The programme has a proactive policy of strengthening health service delivery in the places where clinical research is undertaken, in collaboration with the Ministry of Health, to ensure that research-led services do not seem to provide an undue incentive to join studies, and to build longer term capacity for health care delivery and partnerships for research. For example, through the Ministry of Health collaboration, the research programme supports the provision of medicines, supplies and diagnostic tests to wards in the county hospital and peripheral health facilities where research is conducted alongside care. Kilifi County includes rural and semi-urban populations of around 1 million; subsistence farming is the primary livelihood and between 55% and 65% households live below the poverty line [16]. The majority of residents are from the Mijikenda ethnic group in Kenya [17]; 47% describe Christianity, 13% Islam and 24% traditional beliefs as their faith system. Amongst adults, 45% reported an inability to read a newspaper or letter during randomised household surveys in 2005. This population constitutes the ‘community’ referenced throughout this paper.

### 2.2 Study population, sampling and data collection

Between November 2012 and May 2013, 90 Kilifi residents (Table 1), were engaged in a two-stage consultation process. Community members were drawn from groups of: i) Research staff who were both from, and working directly with, the community: field workers (front-line staff undertaking informed consent processes, interviews and/or sample-taking) and community facilitators [n = 33]; ii) KEMRI community representatives (KCRs, ‘typical’ residents selected by their local communities to support consultation on research-related issues) [18] [n = 22]; iii) Administrative leaders (assistant chiefs) [n = 9]; iv) Opinion leaders (leaders or members of Community Based Organisations, including women's groups, youth groups and Village Dispensary Committees) [n = 22]; and v) mothers of child study participants [n = 4]. Within these groups, participants were purposively selected to maximise diversity, based on criteria of gender, age, education, location (urban or rural) and religion. All participants had at least basic understanding of research and the research institution; opinion leaders through attendance at Open Days (registers of which were used to identify participants for this consultation); KCRs and Assistant Chiefs through training and regular meetings with community liaison staff; and staff through their professional roles and sometimes through basic training on research ethics. Mothers of study participants were chosen by convenience from a non-therapeutic malaria immunology cohort study involving relatively high levels of study benefits. Field workers were selected for their varied experience across different types of research.

**Table 1:** Summary information for participants.

*Role*	*Total number*	*Gender M:F*	*Education range (years)*	*Religion*
Staff: Community facilitators	8	4∶4	12–16 y	Christian 6; Muslim 2
Staff: Field workers	25	17∶8	8–16 y	Christian 25
Assistant chiefs	9	5∶4	12 y	Christian 7; Muslim 2
KEMRI Community Representatives	22	10∶12	1–16 y: 1–8 y (14) 8–12 y (7); College (1)	Christian 15; Muslim 6; Traditional 1
Community based organisation leaders	22	14∶8	0–16 y: 0–8 y (11); 8–12 y (8); college (3)	Christian 16; Muslim 6
Mothers of study children (malaria immunology study)	4	0∶4	0–8 y (3); 12 y (1)	Christian 3; Traditional 1

The structure of the consultation included a half day workshop followed within two weeks by a small group discussion lasting approximately four hours. Five workshops were held, each including between 9 and 31 participants. Each workshop was followed by between 2 and 5 small groups, depending on size; workshops and small groups included people from one of the ‘types’ of participants listed above. Workshops used participatory methods to:

- Share background information on research in a neutral way, drawing on the experience of participants, including information on the nature of research and research review processes;
- Introduce scenarios used throughout the consultation;
- Hold introductory small group and plenary discussions on challenges around levels of benefits and payments that were followed up in later small group discussions.

During workshops, facilitators aimed to explore perceptions of important boundaries to payments and benefits and limit potential influence from introducing value-laden concepts such as ‘undue inducement’ and ‘voluntariness’ at an early stage. Two scenarios, given in Box A, based on current practices in relation to study benefits and payment in the programme, were used as a starting point for discussions: a home-based interview on health-seeking behaviour for childhood fever; and a facility-based Malaria Vaccine Trial.

### Box A: Study scenarios for discussions

#### Scenario 1: A home-based interview on fever treatment-seeking behavior

A field worker visits a home to request that a mother of three young children participate in a study involving a one hour interview on her beliefs about the causes and treatment of fever in children. The mother is offered an opportunity to choose the time for the interview, and the field worker approaches the home respectfully and explains the study carefully.

- Do you think there is a need for this mother to be given anything for her participation in this study? Why do you think this and what should that be?
- What if the interview were longer, say 3 hours?
- What if it included a finger prick sampling of blood?
- If more benefits are seen as important, what role for community vs individual benefits?

#### Scenario 2: Benefits and payments in a Malaria Vaccine Trial

A mother has consented to her child taking part in a Malaria Vaccine trial. This means that it is not known whether this vaccine will work against malaria or not, but the vaccine has been tested in earlier trials and found to be sate and likely to be effective. A blood sample will be taken to test the health of the child before the vaccine is administered; the child will receive a health check after the vaccine is administered; follow up at home for 6 months by a KEMRI Field worker, including 6 visits in total, some of which will include further blood tests; if the child is sick at any time during the study they will receive free treatment; and when needed by the study team, transport will be reimbursed. A study clinician is assigned to the dispensary to attend to all sick children and other community members as a way of giving back to the community.

- What do you think about these benefits and payments?
- Do you think there is a need for this mother to be anything else for her participation in this study? Why do you think this and what should that be?
- What if the trial included adults, not children?
- What differences are there between this situation and Scenario 1?

Subsequent small group discussions supported more in-depth discussion, checking and extending workshop scenarios, ensuring contributions from all participants, and allowing time for reflection between meetings. The method drew on an approach used previously in Kilifi to undertake community consultations on disclosing genetic findings [19]. In contrast to a more typical focus group discussion [20], we built in greater involvement of facilitators in directing the discussion and using probes to support individual and group reflection and debate. Facilitators aimed to explore the views of all participants as far as possible, use non- judgmental probes to explore reasoning and promote reflection, particularly for morally relevant issues, and avoid consensus building, in keeping with substantive forms of deliberative ethics [21], [22]. Discussions were held at venues convenient to participants, in languages of choice (English, Kiswahili or local language) and took approximately 4 hours, including a break for refreshments. Following the usual practices for community engagement activities, non-staff participants were compensated for time spent in these discussions, at a rate of approximately $3.5, along with reimbursement of transport costs.

All authors supported aspects of facilitation in this consultation, with non-staff community group discussions facilitated by MN, FK and SMw. FK and SMw are experienced community facilitators from this community; VM and SMo have been resident in Kilifi for more than 18 years, and MN for 6 years.

### 2.3 Data management and analysis

At workshops, a dedicated note-taker made detailed notes of discussions. At small groups, discussions were recorded, transcribed and translated into English. Translations were undertaken by note-takers present in meetings, experienced staff with fluency in local languages and English, and checked by MN. The study team held debriefings after discussions, using emerging findings to inform on-going topic guide development. Data were managed using Nvivo 10 and Microsoft Word applications, anonymised through coded identities. Analysis used a modified Framework Analysis approach [23], including in-depth reading of transcripts, making detailed summaries of discussions, and developing two sets of analysis charts: i) summaries capturing the range and progression of views in discussions; and ii) detailed charts on themes emerging from text-based analysis of the data and from concepts informing topic guides. Analysis was primarily conducted by MN and VM, with support from all other authors, including an iterative process of cross-checking and discussions around coding of data and development of analysis charts.

### 2.4 Ethical review

The study was approved by the KEMRI Scientific Steering and Ethical Review Committees. This permission included the use of verbal consent for participation in workshops, given the large numbers of people involved in many of the workshops (up to 30) and that these events were very similar in nature to routine non-research community engagement meetings. Verbal consent was documented in workshop minutes. Individual written consent was obtained for participation and voice recording in the subsequent small group discussions. No children or young people (under 18 years) were involved in this study.

## Findings

**Figure 1:**
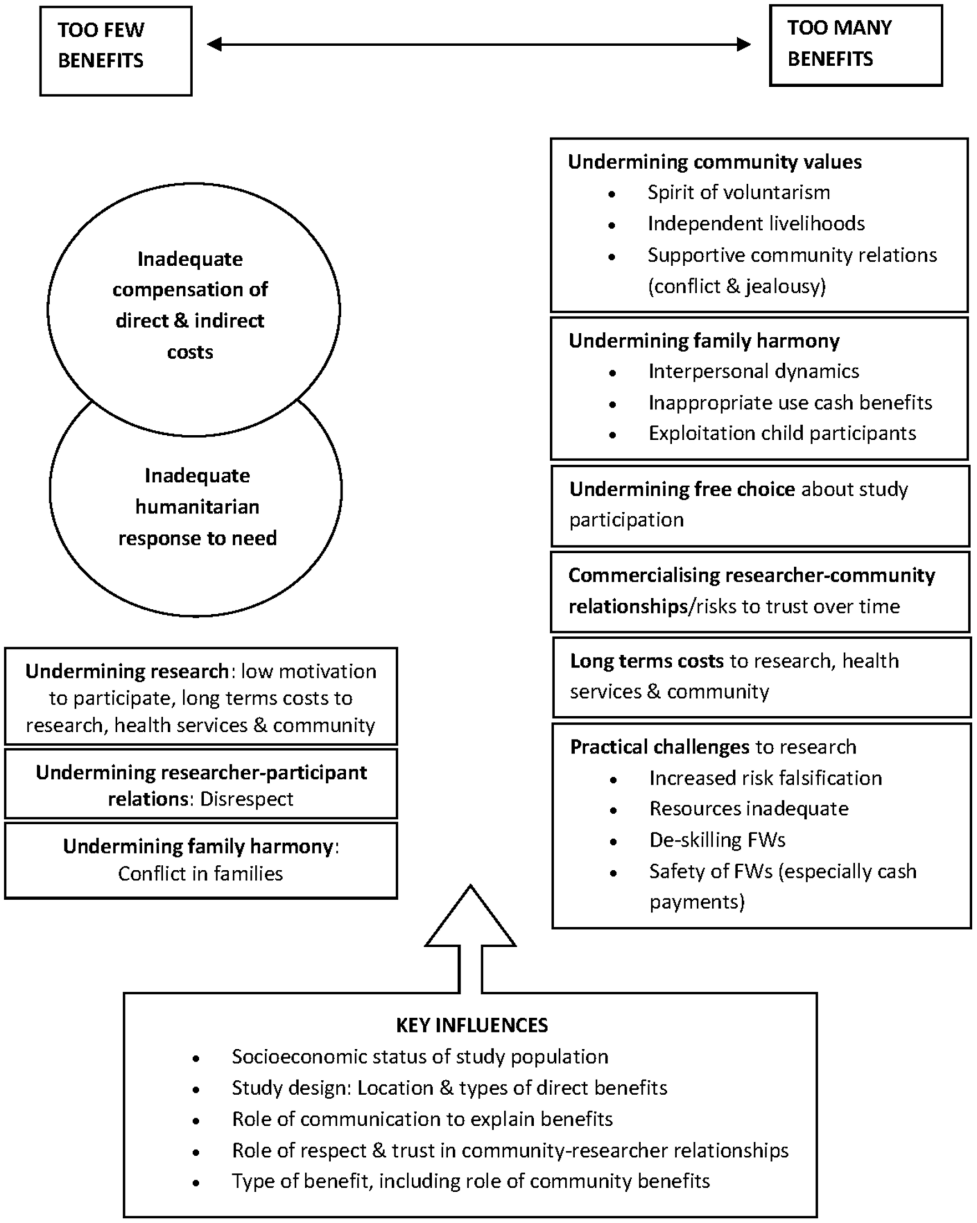
Perceptions of challenges in giving ‘too few’ and ‘too many’ study benefits and payments.

The main focus of the findings are staff and non-staff community members' views on potential challenges when benefits or payments for study participants are set at levels seen as ‘too low or ‘too high’. Although these issues are described separately, in practice there was much interplay between different types of challenges, frequent agreement on their nature, and recognition that the same challenges could result from giving either ‘too few’ or ‘too many’ benefits. Challenges were not described in isolation, but within debates on how to avoid the risks seen for ‘too many’ and ‘too few’ benefits. The major points of difference concerned *the actual financial values at which challenges would be likely to occur* and *how challenges were prioritised* in particular contexts, with greatest diversity and strength of opinion at extremes of both. Throughout, socioeconomic status was perceived as an important influence on the likelihood and magnitude of challenges associated with giving ‘too few’ and ‘too many’ benefits and payments. Figure 1 summarises the findings on types of challenges, described in detail in this section, and highlights important contextual influences, considered in the discussion.

### 3.1 Challenges of giving ‘too many’ benefits

There was clear agreement on the importance of setting upper limits on individual benefits to participants, with a number of often inter-related challenges implicated, as described in the following sections.

#### i. Too much influence on individual decisions

While the inclusion of payments and benefits were seen as a reasonable influence on people's decisions to participate, there was widespread – but not universal - concern about the potential for high levels of benefits to act as a ‘problematic’ form of encouragement. In this situation, the ‘problem’ was mainly related to an unwanted effect on understanding and free choice, often linked to perceptions of the complexity and unfamiliarity of research in this community. People might not think enough about what was involved in participation, but make premature decisions based on an ‘offer’. Alternatively, people who had thought about the study but not fully understood might decide to participate anyway, again based on the ‘offer’.

*…when the benefits are too much …it gets to a point where it's like it's a buy off, you don't have to decide, the price is too good to reject… (Field worker, male, 29 y)*

These concerns were voiced in all groups, but most strongly amongst staff community members. The reason that ‘understanding’ was seen as important often seemed to be related to an intrinsic value or a right to make free decisions:

*So even though there is not really you know taking advantage of the people per se but the fact that it makes people not to think, you know, to consider all the pros and cons of participating in the research, already that's infringing someone's right… (Community facilitator, male)*

Some staff community members also saw understanding as important in supporting cooperation in studies, and - less often – in protecting the safety of participants, who could choose not to participate if they felt risks were too high. In general, issues about safety were not prominent in discussions, a feature likely linked to information given in preliminary workshops about the role of ethical review in checking that risks to participants were reasonable.

In common with many challenges discussed in this consultation, those seen as most at risk of being too easily influenced were the most-poor, thought likely to be most attracted by benefits and payments. At the same time, highlighting debates in the literature, for those who felt that a loss of ‘free choice’ was not particularly important, their reasoning almost always concerned the greater importance for the most-poor of getting high levels of benefits, given the importance of their unmet needs.

#### ii. Undermining household relations

High levels of payments and benefits were also seen as potentially undermining household relations. In this traditionally patrilineal setting [24], male household heads generally are key decision makers in how family resources are used. While there is much diversity in the way household dynamics work in practice, through individual socioeconomic, cultural and temporal influences [25], in many households married women gain access to significant levels of financial resources through their husbands. Giving benefits and payments to mothers of child participants was seen to risk creating family disharmony if levels were ‘too high’. These risks included undermining fathers' traditional control of household resources; generating mistrust between parents; and that fathers taking control of cash benefits might not prioritise the participant child's interests. Concerns about marital disharmony emerged particularly strongly in specific non-staff community groups, but were recognised as a challenge in many others:

*In the norms and traditions of the Mijikendas…men believe that a woman cannot provide things for herself. He believes that he is the sole provider for the woman… when she [mother] gets home the husband might think…it's not… an issue of research but he [KEMRI field worker] had his hidden agenda (Rural assistant chief, female).*

#### iii. Challenges for community relations and values

A common and strongly felt concern about high levels of benefits was the risk of creating conflict within communities, based on jealousy emerging between participants and non-participants [26]. A particular risk was that research selection processes would be seen as biased, leading to rumours of clan preferences:

*But if we look at malaria research, none of my children have suffered from malaria but there is a neighbour whose 2 or 3 children often suffer from that illness… if… its only me who was selected… the person with sick children will feel bad because…hers are always sick, always going to the hospital so why can't she be selected? So then there will be a sort of hatred in that homestead (Rural assistant chief, female).*

In addition, the problem of undermining voluntariness in research was linked by some staff and non-staff community members to the likelihood that people would lose commitment to a spirit of voluntarism, or social cooperation, fundamental to many aspects of community life. The concern was that giving payments and benefits at too high a level to study participants could ‘kill the spirit of volunteering’ by generating an expectation of reward for contributions to community development projects. In this case, it was often felt that any benefits given should act as tokens of appreciation, rather than ‘payment’:

*What I feel is not right is when you get someone used to receiving money. It kills the volunteering spirit and that spirit is very important…When the chief calls us for a meeting….once they find out maize will be distributed a lot of people show up, but if they are told it's [just] a meeting no one comes. So I don't support constant giving of tokens except when one volunteers on their own. [Later] Because of the money [payments to study participants] progress will be hindered (Youth leader, male, 48 y).*

As a further adverse impact on the community, high levels of payments and benefits were viewed as generating the risk of creating a sense of dependency amongst study participants which could, if continued over long periods of time, disrupt economic livelihoods of families and even the wider community (for very large studies). Adding to this risk was the potential for those in receipt of benefits to be looked down upon within the community as people who ‘live on aid’.

#### iv. Impact on the researcher-community relationship

In situations where the level of payments and benefits were seen as ‘too high’, there was a widely perceived risk of creating suspicions about the motivation of researchers and research institutions, and risking breakdown in trust. Where there were pre-existing concerns and rumours about research activities, for example, based on misconceptions about the purpose of research [27] or hidden commercial interests of researchers, giving high levels of benefits and payments was seen as particularly risky for the institution's reputation:

*…giving something like …a plastic mug…depending on what the research has planned… that sounds better to me than giving money because giving money, I tell you, to an African, its directly linked to buying (Field worker, male, 50 y).*

These effects of commercialising the researcher-participant relationship were particularly marked for - but not exclusive to - cash payments. A particularly problematic effect of this ‘transactional’ view of the relationship was seen for research involving children:

*They will not be doing it out of their willingness to participate…they will almost force themselves to participate because they know if their child participates they will benefit. So that will be like selling this child to receive monetary gain and that will be like a business investment (Mother of study child, 40 y, 12 y education).*

One perceived consequence of a shift in the researcher-community relationship towards commercialisation was the setting up of expectations which might be difficult for researchers to meet in future, particularly for organisations with fewer resources. Countering this view, others felt all researchers should be obliged to plan their research with the same reasonable levels of payments and benefits. As a last - and contested - point, a few expressed concerns that commercialising the relationship would take a sense of ownership of the research away from participants and the wider community.

#### v. Negative impacts on research

Three main issues were raised about risks to research through setting benefits and payments at too high a level. Firstly, staff and non-staff community members perceived that people considering participation might falsify information in order to become eligible for studies:

*If people come to know that there are too many benefits…people who do not meet the study criteria will look for any means so as to join… and if she fails to join, she will envy the others who joined (Urban assistant chief, male).*

Secondly, an issue - particularly raised by staff community members - was that over time staff responsible for informed consent would become de-skilled, since a pressure to communicate well would be removed by many people's keenness to participate. Conversely, reasonable levels and forms of benefit to study participants were seen as an appropriate means of supporting the work of this staff group through encouraging recruitment.

Finally, some community members reflected on a risk that giving ‘too many’ benefits would eventually be reflected in the cost and sustainability of research, and therefore the products of research, including that ultimately the vaccines and drugs produced might be unaffordable in low-income countries.

### 3.2 Challenges in giving ‘too few’ benefits

#### i. Unfair economic and financial burdens to families

The risk that ‘too few’ benefits would generate unfair burdens for families was the most serious challenge described in these consultations, and the most strongly and widely expressed concern. Burdens included inadequately taking account of money spent (as a direct cost) and of lost opportunities to earn income or undertake other essential unpaid tasks (indirect costs), including child care, house repairs and subsistence farming [28]. Socioeconomic status was a major influence on the risk of unfairly burdening participants, with potential risks seen as more likely and more serious for the most-poor, but which category included a high proportion of the population overall.

While *direct costs*, for example transport refunds in the Malaria Vaccine Trial scenario, were clearly noticed and more easily accounted for, discussions on the importance and ways of compensating for *indirect costs* involved much more controversy, particularly in accounting for time. There was a common concern about the risk of fundamental forms of unfairness when time was not adequately accounted for on the basis of having unpaid or informal livelihoods:

*… if we're basically saying we are not compensating this person because they… are losing nothing anyway, are we not already taking advantage of their vulnerability in the sense that we know they are not… losing anything and therefore we have no responsibility to give them anything, so we can just take that opportunity to get…that information? (Community facilitator, male)*

Views on the role of time were explored by increasing the length of an interview from 1 to 3 or more hours in research scenario A, and considering time spent at and travelling to a study clinic in Scenario B. The central emerging issue concerned the difficulty in both *noticing* and *accounting* for costs associated with essential unpaid work and lost income, and the uncertain role of time as a parameter in assessing these, particularly given the prevalence of poverty and the nature of subsistence livelihoods:

*[In Kilifi] poverty is the same everywhere. So for that 1 hour…she will think it's better if she had gone to fetch firewood… She might not have anything to cook but she could use firewood to boil water for bathing…a normal person cannot fail to have something to do at home (Christian youth group leader, 29 y, 8 y education)*

Common livelihoods were described as essential unpaid domestic work, subsistence farming and paid casual labour, including piece-meal work (‘*vipande’* in Kiswahili). Time taken from paid work generates a clear indirect cost, but payment in cash or in-kind would also often be involved where a family member or neighbour was asked to take over essential unpaid work. The critical point about compensating for time in relation to these livelihoods was that a *daily* income or food production was often essential to a family's wellbeing, through the ability to ‘put food on the table’ at the end of the day. Indicative of this perception, estimates of the levels of payments and benefits were often focused around resources needed to provide a family meal. Duration of time was generally seen as important; shorter periods of time were seen as less in need of ‘compensation’ because they would be likely to incur less economic costs. For periods of about an hour, many considered that ensuring flexibility (for example, by booking appointments for interviews), giving small tokens of appreciation or simply showing respectful attitudes and communicating carefully would often be enough, assuming participation was truly voluntary. The inclusion of some learning in the interaction would also limit the need for additional ‘benefits’. For periods of time of 3 hours or more, most felt some form of compensation was important, as economic costs would very likely be incurred, with particularly serious implications for the most-poor:

*In my view, it's important that the benefits are increased [with time] because there will still be difficulties that day…even if the transport expenses were refunded, and if they were meant to do some casual work in order to get some food after the visit to the centre, they would probably sleep hungry that day because there is nothing that fills the gap for the day spent at the centre (Christian youth group leader, 29 y, 8 y education)*

Some non-staff community members disagreed, arguing that shorter and longer periods of time have similar requirements for payment or other benefits, particularly for the most-poor where even small sums of money could buy valued goods like a container of water. There were also challenges in using amounts of time (for example hourly rates) as the only parameter of indirect costs of participation. For casual labour, hiring is often linked to availability at key points in time such as the start of the day. Missing the hiring time would exclude the possibility of earning cash that day:

*…especially for the poor families…once you use their time, on humanitarian grounds…even the child may be hungry and then you leave without offering the mother anything. After that the mother goes to look for casual jobs only to be told that there are no vipande remaining and then she goes back home while you still want to participate the child in research, it will be of no help (Village Dispensary Committee member, male 58 y, 12 y education).*

Comparisons were made with social norms in relation to visitors, being described as norms of visitors both giving and receiving. An underlying understanding in relation to these norms was that (within reason) exact amounts of time spent would not be seen as particularly important for visitors, but rather who came and what was discussed:

*OK, with people in our communities… they normally don't weigh the time spent with them. What they'd say is, they came (all laugh) yeah they came…So ok, it doesn't make any difference whether you go there and spend like two, twenty minutes or an hour with them…they don't consider the time…it's your presence there. (Field workers, male, 21 y)*

#### ii. Low motivation to participate & research failure

Since most agreed that payments and benefits were an important motivation to people's decisions to join studies, giving too few benefits was seen as risking research failure. A potential ‘community-wide effect’ was described where word spread that ‘very few benefits’ were associated with a particular study. Staff and non-staff community members were concerned about the knock-on risks of low participation in studies, including failure to develop new treatments and health services and to sustain local employment opportunities:

*… many people will drop out from the study and the study will not succeed… probably you will be laid off, it will be chaos, diseases like malaria will still be there… on the ground people will suffer, they will fall sick…(Urban assistant chief, male)*

More subtly, low levels of motivation might not be very visible to research staff but still have important effects on research and on the researcher-community relationship:

*They will think their time is not used appropriately…you will find you are interviewing her while she continues cleaning utensils, you talk to her but she keeps quiet cleaning her utensils and she bids you goodbye cleaning her utensils!(Laughter) (Rural assistant chief, male)*

A specific concern in relation to risks of low motivation was that while ‘low benefits’ are likely to discourage participation in general, this effect might occur inequitably across a community. This issue was also seen as a form of ‘double unfairness’ in relation to free choice for the most-poor, who might be influenced to participate in studies at levels of payments and benefits that others in the community might even consider exploitative or insulting (that is, being too poor to refuse even a bad offer). Community facilitators described a resulting unfairness in who carries the burden of participation and related risks of bias in research findings that could impact validity.

#### iii. Undermining researcher-community and family relations

As above, where benefits and payments set at ‘too low’ a level appeared insulting, there was a risk of undermining the relationship between researchers and the community, leading to suggestions by staff members that a minimum value should be set:

*… if we were to visit someone and we give him 100 shillings - would they appreciate that? Or you would even create more problems for yourself? Because I think there is a level where you do things in the community and they feel you have actually…undermined them, they wouldn't appreciate it (Community facilitator, male).*

A final point was made about the way that setting benefits and payments at too low a level could have a negative effect on relationships within families, through disagreements on how limited family finances should be prioritised between research and other activities.

## Discussion

Based on a two-stage process of information sharing and guided debate, the study has generated detailed accounts of the experiences, values and reasoned views of community members, highlighting diversity of views, complexity of contextual influences at micro/individual and macro/structural levels, and interplay between the types of risks seen, both in giving ‘too many’ and ‘too few’ benefits and payments. Cross cutting all considerations of study benefits and payments was the need to minimise costs to participants through maximising convenience and flexibility in study planning, and ensuring respectful and skilled communication, including about the purposes of any benefits provided. Many of these findings reflect issues described in the literature on study payments and benefits in the background. In this discussion, we consider the implications of these findings for the ‘undue inducement vs. exploitation’ dilemma and the responsibilities of researchers to respond to structural inequities, and reflect on influences from types of benefit and the history of the research institution in Kilifi.

In doing so, we recognise and have sought to limit potential influences from the authors' positionality throughout the data collection, analysis and interpretation. Similarly the methods used for consultation present potential limitations for the study, in common with many used in qualitative research, that is, that group dynamics, aspects of context and the way in which facilitators share information and moderate discussions may influence participants' contributions. At the same time, we note that debates were generally open and included strong criticism of existing research policy; and that experienced facilitators led the consultations. A further potential influence on participants' contributions was that non staff community stakeholders involved in these discussions were compensated for their time (in this case, for approximately a half day) and given fare reimbursements, as described in the methods section of this paper. Any such potential influence is likely to have been limited by the routine nature of these payments in community engagement activities within the programme, and the focus on research activities throughout the discussions themselves.

### 4.1 The ‘undue inducement vs exploitation’ dilemma

In the background to this paper, we introduced a major concern in the literature that setting study benefits at ‘too high’ a level has the risk of unduly inducing people to participate in research, thereby undermining free choice. While a main counter position in the literature focuses on arguments about the concept of ‘undue’ forms of inducement in research [29], our findings show that this may be one of several challenges in ‘giving too many benefits’. Others include risks to community and family values, and for trust in the community-researcher relationship - particularly for cash benefits. Further, while ‘undue inducement’ has been argued as a largely instrumental challenge in relation to risks to safety of participants [30], many community members in Kilifi saw an intrinsic value in the concept of free choice on the basis of understanding, voicing concerns that ‘too many’ benefits would ‘corrode’ reasoning.

In interpreting these - and other - findings, we reflect on the commonness, strength of feeling and underlying reasons perceived for risks, and their relationship to wider normative accounts in the literature, in keeping with a substantive form of deliberative ethics (Parker 2007). This process included taking account of the existence of local controversy and more general ethical principles that might counter particular views. For example, we described that some community members saw a risk that high levels of benefits could lead to an unwanted form of gender empowerment. The fact that this view was locally controversial and could also be strongly countered by human rights arguments informed our overall analysis of the importance of this risk, while highlighting local sensitivities for researchers. In contrast, at a long standing research institution where much research involves participation of a particular geographic community, we particularly noted risks seen for the quality of researcher-participant relationships. In practice, the attitudes expressed towards this relationship often reflected perceptions of an alignment of long-term interests between researchers and community members, albeit with continuously re-negotiated issues of trust. In other words, while this relationship could be argued as most key to researchers (studies could not be undertaken without community support), individual and community-wide benefits and the social value of research in Kilifi were also highly valued. Similarly, in a community where relationships within extended families and social networks are often seen as intrinsically important, and essential to the welfare of individuals and the community, risks to these structures through generating conflict, jealousy, overdependence and impacts on livelihoods by giving ‘too many’ benefits seem important forms of harm.

But across all these discussions, the most prominent and consistent concern about levels of benefits and payments was the risk of giving ‘too few’ individual study benefits, with heightened risks seen for the most-poor, in a community where poverty was described as widespread. Common informal types of employment in this community do not lend themselves easily to comparisons with hourly or daily rates, and much unpaid activity is essential domestic and farming work. These types of indirect costs are often not obvious and difficult to put a monetary value on. Reflecting this challenge, a common recommendation emerged that benefits should relate to a family's ability to ‘put food on the table that day’. The relatively hidden nature of such economic costs was particularly reflected in discussions of scenario B (the malaria vaccine trial) in this study. While some participants spontaneously noted that providing medical services to participants during research clinic appointments would not compensate for time spent in travelling to and attending the clinic, many only recognised this issue after it had been raised by others. On reflection, it was widely felt within all groups that these *indirect* costs of research participation raised an important potential gap in current practices on study benefits in the programme for some types of research; an issue that has been taken forwards in setting programme policy.

Overall, we heard strong arguments against an over-riding importance of *voluntariness in research* when placed against the *essential support* very poor families might gain from study benefits, including strong views that undermining voluntarism should not be used as an excuse *not* to provide benefits, particularly for the most-poor. This argument was made in ways that reflect two different debates in the literature. The first was the need to provide adequate compensation for indirect costs of participation, coupled with a view that these are hard to determine for the types of livelihoods common in the most-poor. This is a well understood obligation of researchers, strengthened here by the understanding that failing to compensate adequately can lead in practice to substantial forms of harm (such as an inability to provide food for the family). The second was an argued ‘humanitarian’ responsibility of researchers working in communities affected by poverty. This proposal seems to reflect arguments in the literature on the importance of taking account of macro-level inequities and avoiding a ‘thin view’ of what counts as an ethical issue in international research [8], [10], for example in the Fair Benefits debate [12]. This literature also recognises that researchers' potential macro-level responsibilities may be less strong and more limited than those related to micro-level ethical issues, and that a normative account of macro-level responsibilities remains controversial [8]. But, in the reasoned debates in Kilifi, humanitarian arguments not only generated very strong emotions, but were also *very difficult to separate* from fair compensation arguments, given challenges in assessing indirect costs related to informal livelihoods. On the basis of these findings and our reading of the literature, we argue that international researchers working in low-income settings should consider the potential for underestimating indirect costs *as a more likely and serious risk than that of undermining free choice*. In any case, accepting a risk of overestimating indirect costs taps into commitments to recognise researchers' macro-level ethical responsibilities, and occurs at levels more likely to support than undermine ‘free choice’.

As has been suggested in the literature [11], [12], [14], a potentially more substantial way in which researchers can respond to structural inequities in this context is through the provision of community-wide benefits, including strengthening community-wide medical services within studies and across the programme in collaboration with the Ministry of Health. In addition, in this study, some modest calls were made for this response to be extended to other (non-medical) areas of unmet needs in the community, including access to water or school structures. At the same time, we noted that community-wide benefits cannot substitute for individual study benefits, where direct or indirect costs are incurred.

Overall, for both micro and macro-level ethical issues, we take seriously the strength of feeling from many in this community in support of a ‘humanitarian’ response, following Wertheimer's (1996) prescient line that: ‘…the intuitions that some mutually beneficial agreements are unfair is so strong that… it would be quite premature to think that no such principle can be defended.’ [31]. More recently Ballantyne (2008) draws on this quotation in arguing for researchers' responsibilities for macro-level ethical issues; concluding that ‘a normative account of fair distribution in the international research context should now be the primary goal of those who endorse the fair benefits model’ [6]. We support her conclusion, and propose that a normative account might include the challenges in practice of differentiating between *inarguable* responsibilities of researchers in relation to indirect costs and *arguable* ones for ‘humanitarian’ actions, particularly for the group of people at greatest risk of harm and need of assistance, that is, the most-poor.

### 4.2 The role of cash benefits

Across all these potential risks, cash benefits were generally the most controversial, seen as important in promoting individual control over how benefits are realised, but with greater risks than non-cash benefits of generating all the problems associated with ‘too many’ benefits. The clear face value of cash, for example, was seen to facilitate comparisons in ways likely to generate dissatisfaction and conflict. In comparison, giving non-cash benefits was seen as a more familiar and less sensitive form of benefit, and not open to use in less beneficial ways, but importantly risked failing to meet the real needs of participants in practice. We see these perceptions about the potential commercialising effect of cash reflected in the literature, for example, from social psychology where ‘money itself can be a cue to the type of exchange that individuals consider themselves to be in (page 792 [32]).

### 4.3 An influence of institutional history and resources

During these discussions we probed for the potential for features of the research institution and its ways of working to influence discussions, as a long-standing internationally-funded research programme. There were views that an internationally-funded institution should provide higher levels of study benefits than less well-resourced organisations. But this expectation was importantly countered by recognition of the contributions to community-wide health care and local employment the institution has made over a long time, and *hopes that this would continue into the future*. Community members described a form of trust in the research institution that included anticipation of future benefits. This contrasted with the attitudes and expectations for a hypothetical less well-established institution, which could be ‘out for what it can get’ and planning to ‘take and run’. Views on some level of convergence of the *long term interests* between the research institution and the community in Kilifi seemed to underpin willingness to consider study-specific and community-wide benefits in support of both.

## Conclusions

Providing benefits and payments to participants in international research in low-income settings are essential ways for researchers to meet their micro-level ethical responsibilities and may provide a means of addressing macro-level issues of social justice in these contexts. Understanding how these can be achieved requires a careful account of social realities and local judgment. Risks of undermining voluntariness are important constraints on levels of benefits alongside risks to community and family values and undermining trust in researchers, but the risks of inadequately compensating indirect costs may be greater. Indirect costs related to informal livelihoods and essential unpaid work are difficult to assess; and are most common and most compellingly require compensation in the most-poor. Policies based on reasonable but generous estimates of indirect costs and the provision of community-wide benefits, made sustainable through partnerships with government providers, are important ways for international researchers working in low-income settings to respond to macro-level ethical issues, particularly but not exclusively for long term research institutions working in particular geographic communities.
